# Supplementary material for: Heat adaptation of phage T7 under an extended genetic code
Source: Virus Evol. 2021 Dec 1;7(2):veab100. doi: 10.1093/ve/veab100 (PMC8923235; doi:10.1093/ve/veab100)
Supplement: veab100_Supp [file veab100_supp.zip › Sequencing_Analysis.zip!/Sequencing_Analysis/A1/evidence/RA_38.html]

BRESEQ :: Evidence


| Read alignment evidence... | | | | | | | | | | | |
| --- | --- | --- | --- | --- | --- | --- | --- | --- | --- | --- | --- |
|  | seq id | position | | ref | new | freq | score (cons/poly) | reads | annotation | genes | product |
| \* | NC\_001604.1 | 33,022 | 0 | G | T | 43.2% | 3195.7 / inf | 3683 | intergenic (–/–) | *–/–* | –/– |
| Reads supporting (aligned to +/- strand):  **ref** base G (1200/889);  **new** base T (914/672);  **total** (2120/1563) | | | | | | | | | | | |
| Fisher's exact test for biased strand distribution *p*-value = 9.20e-01 | | | | | | | | | | | |
| Kolmogorov-Smirnov test that lower quality scores support variant *p*-value = 1.59e-04 | | | | | | | | | | | |

Only 100 of 3689 total aligned reads displayed.  

`GATGAGATGCTTAAGGAATTACACGGGGTGAAGGAAGTAACACCAGAGATGGTAGAGAAGTACGCTATGGATAAGGCTTATGGTATCTCCCACTCAGACCAGTTCACCAACAGTTCCATAATAGAAGAGAACATTGAGGGCTTAGTAGGTATCGAGAATAACTCATTCCTTGAGGCACGTAACTTGTTTGATTCGGACCTATCCATCACTATGCCAGACGGACAGCAATTCTCAGTGAATGACCTAAGGGACTTCGATATGTTCCGCATCATGCCAGCGTATGACCGCCGTGTCAATGGTGACATCGCCATCATGGGGTCTACTGGTAAAACCACTAAGGAACTTAAGGATGAGATTTTGGCTCTCAAAGCGAAAGCTGAGGGAGACGGTAAGAAGACTGGCGAGGTACATGCTTTAATGGATACCGTTAAGATTCTTACTGGTCGTGCTAGACGCAATCAGGACACTGTGTGGGAAACCTCACTGCGTGCCATCAATGACCTAGGGTTCTTCGCTAAGAACGCCTACATGGGTGCTCAGAACATTACGGAGATTGCTGGGATGATTGTCACTGGTAACGTTCGTGCTCTAGGGCATGGT  >  NC_001604.1/32722‑33321`  

                                                                                                                                                                                                                                                                                                            `|`                                                                                                                                                                                                                                                                                                             
   `tgtataagagacagaTTACACGGGGTGAAGGAAGTAACACCAGAGATGGTAGAGAAGTACGCTATGGATAAGGCTTATGGTATCTACAACTCAGACCAGTTCACCAACAGTTCCATAATAGAAGAGAACATTGAGGGCTTAGTAGGTATCGAGAATAACTCATTCCTTGAGGCACGTAACTTGTTTGATTCGGACCTATCCATCACTATGCCAGACGGACAGCAATTCTCAGTGAATGACCTAAGGGACTTCGATATGTTCCGCATCATGCCAGCGTATGACCGCCGTGTCAATGGTGACa                                                                                                                                                                                                                                                                                                          <  1:142246/287‑1 (MQ=255)`  
    `agaTGCGTAAGGAATTACACGGGGTGAAGGAAGTAACACCAGAGATGGTAGAGAAGTACGCTATGGATAAGGCTTATGGTATCTCCCATTCAGACCAGTTCACCAACAGTTCCATAATAGAAGAGAACATTGAGGGCTTAGTAGGTATCGAGAATAACTCATTCCTTGAGGCACGTAACTTGTTTGATTCGGACCTATCCATCACTATGCCAGACGGACAGCAATTCTCAGTGAATGACCTAAGGGACTTCGATATGTTCCGCATCATGCCAGCGTATGACCGCCGTGTCAAGGGTGACAt                                                                                                                                                                                                                                                                                                         <  1:32470/301‑1 (MQ=255)`  
        `gCTTAAGGAATTACACGGGGTGAAGGAAGTAACACCAGAGATTGTAGAGAAGTACGCTATGGATAAGGCTTATGGTATCTCCCACTCAGACCAGTTCACCAACAGTTCCATAATAGAAGAGAACATTGAGGGCTTAGTAGGTATCGAGAATAACTCATTCCTTGAGGCACGTAACTTGTTTGATTCGGACCTATCCATCACTATGCCAGACGGACAGCAATTCTCAGTGAATGACCTAAGGGACTTCGATATGTTCCGCATCATGCCAGCGTATGACCGCCGTGTCAATGGTGACATCGcc                                                                                                                                                                                                                                                                                                     <  1:177478/301‑1 (MQ=255)`  
        `gCTTAAGGAATTACACGGGGTGAAGGAAGTAACACCAGAGATGGTAGAGAAGTACGCTATGGATAATGCTTATGGTATCTCCCACTCAGACCAGTTCACCAACAGTTCCATAATAGAAGAGAACATTGAGGGCTTAGTAGGTATCGAGAATAACTCATTCCTTGAGGCACGTAACTTGTTTGATTCGGACCTATCCATCACTATGCCAGACGGACAGCAATTCTCAGTGAATGACCTAAGGGACTTCGATATGTTCCGCATCATGCCAGCGTATGACCGCCGTGTCAATGGTTACATCGcc                                                                                                                                                                                                                                                                                                     <  1:434131/301‑1 (MQ=255)`  
                 `aTTACACGGGGTGAAGGAAGTAACACCAGAGATGGTAGAGAAGTACGCTATGGATAAGGCTTATGGTATCTCCCACTCAGACCAGTTCACCAACAGTTCCATAATAGAAGAGAACATTGAGGGCTTAGTAGGTATCGAGAATAACTCATTCCTTGAGGCACGTAACTTGTTTGATTCGGACCTATCCATCACTATGCCAGACGGACAGCAATTCTCAGTGAATGACCTAAGGGACTTCGATATGTTCCGCATCATGCCAGCGTATGACCGCCGTGTCAATGGTTACATCGCCATCATgggg                                                                                                                                                                                                                                                                                            <  1:266109/301‑1 (MQ=255)`  
                         `gggTGAAGGAAGTAACACCAGAGATGGTAGAGAAGTACGCTATGGATAAGGCTTATGGTATCTCCCACTCAGACCAGTTCACCAACAGTTCCATAATAGAAGAGAACATTGAGGGCTTAGTAGGTATCGAGAATAACTCATTCCTTGAGGCACGTAACTTGTTTGATTCGGCCCTATCCATCACTATGCCAGACGGACAGCAATTCTCAGTGAATGACCTAAGGGACTTCGATATGTTCCGCATCATGCCAGCGTATGACCGCCGTGTCAATGGTGACATCGCCATCATGGGGTCTACTgg                                                                                                                                                                                                                                                                                    >  1:254051/1‑301 (MQ=255)`  
                             `gaaggaagTAACACCAGAGATGGTAGAGAAGTACGCTATGGATAAGGCTTATGGTATCTCCCACTCAGACCAGTTCACCAACAGTTCCATAATAGAAGAGAACATTGAGGGCTTAGTAGGTATCGAGAATAACTCATTCCTTGAGGCACGTAACTTGTTTGATTCGGACCTATCCATCACTATGCCAGACGGACAGCAATTCTCAGTGAATGACCTAAGGGACTTCGATATGTTCCGCATCATGCCAGCGTATGACCGCCGTGTCAATGGTGACATCGCCATCATGGGGTCTACTGGTaaa                                                                                                                                                                                                                                                                                >  1:146659/1‑301 (MQ=255)`  
                                   `agTAACACCAGAGATGGTAGAGAAGTACGCTATGGATAAGGCTTATGGTATCTCCCACTCAGACCAGTTCACCAACAGTTCCATAATAGAAGAGAACATTGAGGGCTTAGTAGGTATCGGGAATAACTCATTCCTTGAGGCACGTAACTTGTTTGATTCGGACCTATCCATCACTATGCCAGACGGACAGCAATTCTCAGTGAATGACCTAAGGGACTTCGATATGTTCCGCATCATGCCAGCGTATGACCGCCGTGTCAATGGTGACATCGCCATCATGGGGTCTACTGGTAAAACCACt                                                                                                                                                                                                                                                                          >  1:100157/1‑301 (MQ=255)`  
                                       `gcacCAGGGATGGTAAAAAAGTACGCTATGCATAAGGCTTAAGGTATCTCACACACAGACCAGTTCACCAACAGTTCCATAATAGCAGAGAACATTGAGGGCTTAGTAGGTATCGAGAATAACTCATTCCTTGAGGCACGTAACTTGTTTGATTCGGACCTATCCATCACTAGGCCAGACGGACAGCAATTCTCAGTGAATGACCTAAGGGACTTCGATATGTTCCGCATCATGCCAGCGTATGACCGCCGTGTCAATGGTGACATCGCCATCATGGGGTCTACTGGTAAAACCACTAAgg                                                                                                                                                                                                                                                                      <  1:74672/300‑1 (MQ=255)`  
                                             `gataTGGTAGAGAAGTACGCTATGGATAAGGCTTATGGTATCTCCCACTCAGACCAGTTCACCAACAGTTCCATAATAGAAGAGAACATTGAGGGCTTAGTAGGTATCGAGAATAACTCATTCCTTGAGGCACGTAACTTGTTTGATTCGGACCTATCCATCACTATGCCAGACGGACAGCAATTCTCAGTGAATGACCTAAGGGACTTCGATATGTTCCGCATCATGCCAGCGTATGACCGCCGTGTCAATGGTGACATCGCCATCATGGGGTCTACTGGTAAAACAACTAAGGAACTTa                                                                                                                                                                                                                                                                >  1:299511/4‑301 (MQ=255)`  
                                               `gaTGGGAGAGAAGTACGCTATGGATAAGGCTTATGGTATCTCCCACTCAGACCAGTTCACCAACAGTTCCATAATAGAAGAGAACATTGAGGGCTTAGTAGGTATCGAGAATAACTCATTCCTTGAGGCACGTAACTTGTTTGATTCGGACCTATCCATAACTATGCCAGACGGACAGCAATTCTCAGTGAATGACCTAAGGGACTTCGATATGTTCCGCATCATGCCAGCGTATGACCGCCGTGTCAATGGTGACATCGCCATCATGGTGTCTACTGGTAAAACCACTAAGGAACTTaat                                                                                                                                                                                                                                                              <  1:368764/301‑2 (MQ=255)`  
                                                   `gTAGAGAAGTACGCTATGGATAAGGCTTATGGTATCTACCACTCAGACCAGTTCACCAACAGTTCCATAATAGAAGAGAACATTGAGGGCTTAGTAGGTATCGAGAATAACTCATTCCTTGAGGCACGTAACTTGTTTGATTCGGACCTCTCCATCACTATGCCAGACGGACAGCAATTCTCAGTGAATGACCTAAGGGACTTCGATATGTTCCGCATAATGCCAGCGTATGACTGCCGTGTCAATGGATACATCGCCATCATGGGGTCTACTGGTAAAACAACTATGGAACTTacgctgt                                                                                                                                                                                                                                                          >  1:115495/1‑295 (MQ=255)`  
                                                       `agatgagtataagagacagggCTTATGGTATCTCCCACTACGACCAGTTCACCAAAAGTTCCATAAGAGAAGAGAACAATGAGGGCTTAGTAGGGATCGAGAATAACTCATTCCTTGAGGCACGTAACTTGTTTGATTCGGACCTATCCATCACTATGCCAGACGGACAGCAATTCTAAGTGAATTACCTAAGGGACTTCGCTATGTTCCGAATCATGCCAGCGTATGACCGCCGGGTCAATTGTGACATCGCCATCATGGGGTCTACTGGTAAAACCACTAAGGAACTTAATGATGAGAt                                                                                                                                                                                                                                                      <  1:329604/282‑1 (MQ=255)`  
                                                        `gaAGTACGCTATGGATAAGGCTTATGGTATCTCCCACTCAGACCAGTTCACCACAAGTTCCATACTAGAAGAGCACATTGAGGGCTTAGTAGGTATCGCGAATAACTCATTCCTTGAGGCACGTCACTTGTTTGATTCGGACCTATCCATCACTATGCCAGACGGACAGCAATTCTCAGTGACTGACCTAAGGGACTTCGATATGTTCCGCATCATGCCAGCGTATGACCGTCGTGTCCATGGTGACATCGCAATCATGGGGTCTACTGGTACACCCACTCAGCACCTTAACGTTGAGCtt                                                                                                                                                                                                                                                     >  1:364455/1‑301 (MQ=255)`  
                                                        `gaAGTACGCTATGGATAAGGCTTATGGTATCTCCCACTCAGACCAGTTCACCAACAGTTCCATAATAGAAGAGAACATTGAGGGCTTAGTAGGTATCGAGAATAACTCATTCCTTGAGGCACGTAACTTGTTTGATTCGGACCTATCCATCACTATGCCAGACGGACAGCAATTCTCAGTGAATGACCTAAGGGACTTCGATATGTTCCGCATCATGCCAGCGTATGACCGCCGTGTCCATGGTGACATCGCCATCATGGAGTCTACTGGTAAAACCACTAAGGAACTTAATGATGCGAtt                                                                                                                                                                                                                                                     >  1:361406/1‑301 (MQ=255)`  
                                                           `gTACGCTATGGATAAGGCTTATGGTATCTCCCACTCAGACCAGTTCACCAACAGTTCCATAATAGAAGAGAACATTGAGGTCTTAGTAGGTATCGAGAATAACTCATTCCTTGAGGCACGTAACTTGTTTGATTCGGACCTATCCATCACTATGCCAGACGGACAGCAATTCTCAGTGAATGACCTAAGGGACTTCGATATGTTCCGCATCATGCCAGCGTCTGACCGCCGTGCCCATGGTTACATCGCCATCATGGGGTCTACTGTTACAACCACTAAggctgtctcttatacacatctc                                                                                                                                                                                                                                                  >  1:356702/1‑281 (MQ=255)`  
                                                                  `aTGGATAAGGCTTATGGTATCTCCCACTCAGACCAGTTCACCATCAGTTCCATAATAGAAGATAACATTAAGGGCTTAATAGGTATCGAGAATAACTCATTCCTTGAGGCACGTAACTTGTTTGATTCGGACCTATCCATCACTATGCCAGACGGACAGCAATTCTCAGTGAATGACCTAAGGGACTTCGATATGTTCCGCATCATGCCAGCGTATGACCGCCGTGTCAATGGTGACATCGCCATCATGGGGTCTACTGGTAAAACCACTAAGGAACTTACTGATGAAATTTTGGctctac                                                                                                                                                                                                                                           >  1:45845/1‑299 (MQ=255)`  
                                                                    `ggATAAGGCTTATGGTATCTCCCACTCAGACCAGTTCACCAACAGTTCCATAATAGAAGAGAACATTGAGGGCTTAGTAGGTATCGAGAATAACTCATTCCTTGAGGCACGTAACTTGTTTGATTCGGACCTATCCATTACTATGCCAGACGGACAGCAATTCTCAGTGAATGACCTAAGGGACTTCGATATGTTCCGCATCATGCCAGCGTATGACCGCCGTGTCAATGGTTACATCGCCATCATGGGGTCTACTGGTAAAACCACTAAGGAACTTAAGGATTAACTTTTGGCTCTCaca                                                                                                                                                                                                                                         >  1:387388/1‑299 (MQ=255)`  
                                                                    `ggATAAGGCTTATGGTATCTCCCACTCAGACCAGTTCACCAACAGTTCCATAATAGAAGAGAACATTGAGGGCTTAGTAGGTATCGAGAATAACTCATTCCTTGAGGCACGTAACTTGTTTGATTCGGACCTATCCATCACTATGCCAGACGGACAGCAATTCTCCGTGAATGAACTAAGGGACTTCGATATGTTCCGCATCATGCCAGCGTATGACCGCCGTGTCAATGGTTACATCGCCATCATGGGGTCTACTGATAAAACCACTAAGGAACTTAAGGATGAGATTTTGGCGCTCaaa                                                                                                                                                                                                                                         >  1:90230/1‑301 (MQ=255)`  
                                                                    `ggATAAGGCTTATGGTATCTCCCACTCAGACCAGTTCACCAACAGTTCCATAATAGAAGAGAACATTGAGGGCTTAGTAGGTACCGAGAATAACTCATTCCTTGAGGCACGTAACTTGTTTGATTCGGACCTATCCATCACTATGCCAGCCGGACAGCAATTCTCAGTGAATGACCTAAGGGACTTCGATATGTTCCGCATCATGCCAGCGTATGACCGCCGTGTCAATGGTGACATCGCCATCATGGGGTCTACTGGTAAAACCACTAAGGAACTTAATGATGAGATTTTGGCTCTCaaa                                                                                                                                                                                                                                         >  1:474675/1‑301 (MQ=255)`  
                                                                          `ggCTTATGGTATCTCCCACTCAGACCAGTTGACCAACAGTTCGATACTAGAAGAGAACATTGAGGGCGTAGGATGTAGCGAGAATAACTCATTCCTTGAGGCACGTAACTTGTTTGATTCGGACCGATCCATCACTATGACAGACCGACCACAATTCTCAGTGCATGACCGGAAGTACTTCTATATGGTGCGCATCTTGCAAGCGTATGACCGACGTGTCTCTGGGTACATCGCCATCGTGGGGTCTACTGGTAAAACCCCTAAGGCACTGAAGGACGAGATTTTGGCCCTCACAGCGaaa                                                                                                                                                                                                                                   >  1:495666/1‑301 (MQ=255)`  
                                                                          `ggCTTATGGTATCTCCCACTCAGACCAGTTCACCAACAGTTCCATAATAGAAGAGAACATTGAGGGCTTAGTAGGTATCGAGAATAACTCATTCCTTGAGGCACGTAACTTGTTTGATTCGGACCTATCCATCACTATGCCAGACGGACAGCAATTCTCAGTGAATGACCTAAGGGACTTCGATATGTTCCGCATCATGCCAGCGTATGACCGCCGTGTCAATGGTTACATCGCCATCATGGGGTCTACTGGTAAAACCACTAAGGAACTTAAGGATGAGATTTTGGCTCTCAAAGCGaaa                                                                                                                                                                                                                                   >  1:348011/1‑301 (MQ=255)`  
                                                                               `aTGGTATCTCCAACTCAGACCAGTTCACCAACAGTTCCATAATAGAAGAGAACATTGAGGGCTTAGTAGGTATCGAGAATAACTCATTCCTTGAGGCACGTAACTTGTTTGATTCGGACCTATCCATCACTATGCCAAACGGACAGCAATTCTCAGTGAATGACCTAAGGGACTTCGATATGTTCCGCATCATGCCAGCGTATGACCGCCGTGTCAATGGTGACATCGCCATCATGGGGTCTACTGGTAAAACCACTAAGGAACTTAAGGATGAGATTTTGGCTATCAAAGCGAAAGCTGa                                                                                                                                                                                                                              >  1:240188/1‑301 (MQ=255)`  
                                                                                 `ggTATCTCCCACTCAGACCAGTTCACCAACAGTTCCATAATAGAAGAGAACATTGAGGGCTTAGTAGGTATCGAGAATAACTCATTCCTTGAGGCCCGTAACTTGTTTGATTCGGACCTATCCATCACTATGCCAGACGGACAGCAATTCTCAGTGAATGACCTAAGGTCCTTCGATATGTTCCGCATCATGCCAGCGTATGACCGCCTTGTCACTGGTTACATCGCCATCATGGTGTCTACTGGTAAAACCACTAAGGAACTTAAGGCTGAGATTTTGGCTCTCAAATCGAAAGCTGCgg                                                                                                                                                                                                                            >  1:168610/1‑301 (MQ=255)`  
                                                                                  `acacctcCCACTCAGACCCGTTCACAAACAGTTCCATACTAGAAGAGAACATTGAGGTCTTAGTAGGTTTCGAGAATAACTCATTCCTTGAGGCACGT‑ACTTGTTTGATTCGGACCTATCCATCACTATGCCAGACGGACAGCAATTCTCAGTGAATGACCTAAGGGACTTCGATATGTTCCGCATCATGCCAGCGTATGACCGCCGTGTCAATGGTTACATCGGCATCATGGGGTCTACTGGTAAAACCACTAAGGAACTTAAGGATGAGATTTTGGCTCTCAAAGCGAAAGCTGAGGga                                                                                                                                                                                                                          <  1:204996/297‑1 (MQ=255)`  
                                                                                    `atgacaaactcaagccaaatccacAACAGTTCCATAAAAAAAGAGAAACTTGAAGGCTTAGTAAGGAATGAGAAAAACCCATTCCTTTGGGCACGTAACTTGTTTGATTCGGACCTATCCATCACTATGCCAGACGGACAGCAATTCTCAGTGAATGACCTAAGGGACTTCGATATGTTCCGCATCATGCCAGCGTATGACCGCCGTGTCAATGGTTACATCGCCATCATGGGGTCTACTGGTAAAACCACTAAGGAACTTAAGGATGAGATTTTGGCTCTCAAAGCGAAAGCTGAGGgag                                                                                                                                                                                                                         <  1:128726/278‑1 (MQ=255)`  
                                                                                    `aTCTCCCACTCAGACCAGTTCACCAACAGTTCCATAATAGAAGAGAACATTGAGGGCTTAGTAGGTATCGAGAATAGCTCATTCCTTGAGGCACGTAACTTGTTTGATTCGGACCTATCCATCACTATGCCAGACGGACAGCAATTCTCAGTGAATGACCTAAGGGACTTCGATATGTTCCGCATCATGCCAGCGTATGACCGCCGTGTCAATGGTTACATCGCCATCATGGGGTCTACTGGTAAAACCACTAAGGAACTTAAGGATGTGATTTTGGCTCTCAAAGCGAAAGCTGAGGgaa                                                                                                                                                                                                                         >  1:150956/1‑300 (MQ=255)`  
                                                                                     `tctcCCACTCAGACCAGTTCACCAACAGTTCCACAATAGAAGAGAACATTGAGGGCTTAGTAGGTATCGAGAATAACTCATTCCTTGAGGCACGTAACTTGTTTGATTCGGACCTATCCATCACTATGCCAGACGGACAGCAATTCTCAGTGAATGACCTAAGGGACTTCGATATGTTCCGCATCATGCCAGCGTATGACCGCCGTGTCAATGGTGACATCGCCATCATGGGGTCTACTGGTAAAACCACTAAGGAACTTAAGGATGAGATTTTGGCTCTCAAAGCGAAAGCTGAGGgaga                                                                                                                                                                                                                        >  1:218543/1‑301 (MQ=255)`  
                                                                                      `ctcCCACTCAGACCAGTTCACCAACAGTTCCATAATAGAAGAGAACATTGAGGGCTTAGTAGGTATCGAGAATAACTCATTCCTTGAGGCACGTAACTTGTTTGATTCGGACCTATCCATCACTATGCCAGACGGACAGCAATTCTCAGTGAATGACCTAAGGGACTTCGATATGTTCCGCATCATGCCAGCGTATGACCGCCGTGTCAATGGTTACATCGCCATCATGGGGTCTACTGGTAAAACCACTAAGGAACTTAAGGATGAGATTTTGGCTCTCAAAGCGAAAGCTGAGGGAGAc                                                                                                                                                                                                                       >  1:480044/1‑301 (MQ=255)`  
                                                                                      `ctcCCACTCAGACCAGTTAACCAACAGTTCCATAATAGAAGAGAACATTGAGGGCTTAGTAGGTATCGAGAATAACTCATTCCTTGAGGCACGTAACTTGTTTGATTCGGACCTATCCATCACTATGCCAGACGGACAGCAATTCTCAGTGAATGACCTAAGGGACTTCGATATGTTCCGCATCATGCCAGCGTATGACCGCCGTGTCAATGGTTACATCGCCATCATGGGGTCTACTGGTAAAACCACTAAGGAACTTAAGGATGAGATTTTGGCTCTCAAAGCGAAAGCTGAGGGAGAc                                                                                                                                                                                                                       <  1:401599/301‑1 (MQ=255)`  
                                                                                            `cTCAGACCAGTTCACCAACAGTTCCATAATAGAAGAGAACATTGAGGGCTTAGTAGGTATCGCGAATAACTCATTCCTTGAGGCACGTAACTTGTTTGATTCGGACCTATCCATCACTATGCCAGACGGACAGAAATTCTCAGTGAATGACCTAAGGGACTTCGCTATGTTCCGCATCATGCCAGCGTATGACCGCCGTGTCAATGGTGACATCGCCATCATGGGGTCTACTGGTACAACCACTAAGGAACTTAATGATGAGATTTTGGCTCTAAACGCGAAAGCTGAGGGAGACGGTaaa                                                                                                                                                                                                                 >  1:309494/1‑300 (MQ=255)`  
                                                                                             `tCAGATCAGTTCACCAACAGTTCCATAATAGAAGTGAACATTGAGGGCTTAGTAGGTATCGAGAATAACTCATTCCTTGAGACACGTAACTTGTTTGATTCGGACCTATCCATCACTATGCCAGACGGACAGCAATTCTCAGTGAATGACCTAAGGGACTTCGATATGTTCCGCATCATGCCAGCGTATGACCGCCGTGTCAATGGTGACATCGCCATCATGGGGTCTACTGGTAAAAACACTAAGGAACTTAAGGATGAGATTTTGGCTCTCAAAGCGAAAGCTGAGGGAGACGGTaaga                                                                                                                                                                                                                <  1:283719/301‑1 (MQ=255)`  
                                                                                                `atatacagcACCAAAAGTGCCATACTATAATAGAACATGGAGTGCTGGTTAGGGAGCTAGAATAACGCATTCCGTGAGGCACGTAACTTGATGGATTCGGACCTATCCATCACTATGCCAGACGGACAGCAATTCTAAGTGAATTACCTAAGGGACTTCGATATGTTCCGCATCATGCCAGCGTATGACCGCCGTGTCAATGGTTACATCGCCATCATGGGGTCTACTGGTAAAACCACTAAGGAACTTAAGGATGAGATTTTGGCTCTCAAAGCGAAAGCTGAGGGAGACGGTAagaaga                                                                                                                                                                                                             <  1:244822/293‑1 (MQ=255)`  
                                                                                                 `aCCAGTGAACCAACAATACAAGAATAGAAGAGAAAAATGAGGGATTAGTAAGAATAGAGAATAACTCATGCCTTGAGGATCGTGACTTATGTGATGAGGACCGATACAGAGCTATGCCAGACGAACAACAATGCTCAGTGAAAGACCTAAGGGAATTGGATATGTGCCGCATCATGCCAGCGTAAGGCCGCCGTGTGAATGGTTACATCGCCATCGTGGGGTCTAGTGGGAAAACCACGAAGTAACTTAAGGATGAGATTTTGGCTCTCAAAGCGAAAGCTGAGGGAGACGGTAAGAAGAc                                                                                                                                                                                                            <  1:418075/301‑1 (MQ=255)`  
                                                                                                     `gTTCACCAACAGTTCCAGAATAGAAGAGAACATTGAGGGCTTAGTAGGTATCGAGAATAACTCATTCCTTGAGGCACGTAACTTGTTTGATTCGTACCTATGAACAACTATGCCAGACGGACAGCAATTTGAAGTGAATGAACTAAGGGCCTTCGATATGTTCCGCCTCATGCCAGCGTATGACCGCCGTTTCAAGGGTGAAATCGCCATCATGGGGTCTCCTGGTAAAACCACTAAGCAACTTAAGGATGAGGTTTTGGCACTCAAAGCGAAAGCGGATGACAACGGTaagactgtctct                                                                                                                                                                                                        >  1:79773/1‑293 (MQ=255)`  
                                                                                                          `ccAACAGTTCCATAATAGAAGAGAACATTGAGGGCTTAGTAGGTATCGAGAATAACTCATTCCTTGAGGCACGTAACTTGTTTGATTCGGACCTATCCATCACTATGCCAGACGGACAGCAATTCTCAGTGAATGACCTAAGGGACTTCGATATGTTCCGCATCATGCCAGCGTATGACCGCCGTGTCAATTGTGACATCGCCATCATGGGGTCTACTGGTAACACCACTAAGGAACTTAAGGATGAGATTTTGGCTCTCAAAGCGAAAGCTGAGGGAGACGGTAAGAAGACTGGCgcatg                                                                                                                                                                                                   >  1:250142/1‑297 (MQ=255)`  
                                                                                                                 `aaaagaacaggaagaGAACATGGAGGGCTTAGTATGTATCGAGAATAACTAATTCCTGGAGGCACGTAACTTGTATGATTCGGACCTATCCATCACTATGCCAGACGGACAGCAATTCTCAGTGAATTACCTAAGGGACTTAGATATGTTCCGAAGAATGCCAGCGTATGACCGCCGTGTCAATGGTTACATCGCCATCAGGGGGTCTACTGGTAAAACCACTAAGGAACTTAAGGATGAGATTTTGGCTCTCAAAGCGAAAGCTGAGGGAGACGGTAAGAAGACTGGCGAGGTACATGCt                                                                                                                                                                                            <  1:54905/291‑1 (MQ=255)`  
                                                                                                                      `taataGAAGAGAACATTGAGGGCTTAGTAGGTATCGAGAATAACTCATTCCTTGAGGCACGTAACTTGTTTGATTCGGACCTATCCATCACTATGCCAGACGGACAGCAATTCTCAGTGAATGACCTAAGGGACTTCGATATGTTCCGCATCATGCCAGCGTATGACCGCCGTGTCAATGGTGACATCGCCATCATGGGGTCTACTGGTAAAACCACTAAGGAACTTAATGATGAGATTTTGGCTCTCACAGCGAAAGCTGAGGGAGACGGTAAGAAGACTGGCGAGGTACATGCTTTAAt                                                                                                                                                                                       >  1:59785/1‑301 (MQ=255)`  
                                                                                                                        `gatgtgtataagagacaGGGCTTAGTAGGTATCGAGAATAACTCATTCCTTGAGGCACGTAACTTGTTTGATTAGGACCTATCCATCACTATGCCAGACGGACAGCAATTCTCAGTGAATGACCTAAGGGACTTCGATATGTTCCGCATCATGCCAGCGTATGACCGCCGTGTCAATGGTTACATCGCCATCATGGGGTCTACTGGTAAAACCACTAAGGAACTTAAGGATGAGATTTTGGCTCTCAAAGCGAAAGCTGAGGGAGACGGTAAGAAGACTGGCGAGGTACATGCTTTAATgg                                                                                                                                                                                     <  1:425585/285‑1 (MQ=255)`  
                                                                                                                            `aagaGAACATTGAGGGAATAGTAGGTATCGAGAATAACTAATTCCGTGAGGAAAGTAACTTGTGTGATTAGGAAAGATCAAACACTATGACAGACGGACAGCAATTCTCAGAGAATGACCGAAGGGACATCGATATGTTCGGCATAAGGCCAGAGTAGGACCGCCGTGTGAATGGTGACATCGAAAGCATGGGGTAAACTGGTAAAACCACAAAGGAACTTAAAGATGAGATTTTGGCTCTCAAAGCGAAAGCTGAGGGAGACGGTAAGAAGACTGGCGAGGTACATGCTTTAATGGATAc                                                                                                                                                                                 <  1:466776/301‑1 (MQ=255)`  
                                                                                                                                   `tataagagacagagtagGTATCGAGAATAACTCATTCCTTGAGGCACGTAACTTGTTTGATTCGGACCTATCCATCACTATGCCAGACGGACAGCAATTCTCAGTGAATGACCTAAGGGACTTCGATATGTTCCGCATCATGCCAGCGTATGACCGCCGTGTCAATGGTGACATCGCCATCATGGGGTCTACTGGTAAAACCACTAAGGAACTTAATGATGAGATTTTGGCTCTCAAAGCGAAAGCTGAGGGAGACGGTAAGAAGACTGGCGAGGTACATGCTTTAATGGATACCATTAAg                                                                                                                                                                          <  1:429605/289‑1 (MQ=255)`  
                                                                                                                                    `tttGTGGGCTTAGTAGGTATCTAGAAAAACTCATTCCTTGATACACGTAACTTGTTTGATTCGGACCTATCCATCACTATGCCAGACGGACCTCAATTCTCAGTGAATGACCTAAGGGACTTCGATATGTTCCGCATCATGCCAGCGGATGACCGCCGTGTCAATGGTGACATCGCCATCATGGGGTCTACTGGTAAAACCACTAAGGAACTTAATGATGAGATTTTGGCTCTCAAAGCGAAAGCTGAGGGAGACGGTAAGAAGACTGGCGAGGTACATGCTTTAATGGATACCGTTAAGa                                                                                                                                                                         <  1:352701/300‑1 (MQ=255)`  
                                                                                                                                          `ggCTTAGTAGGTATCGAGAATAACTCATTCCTTGAGGCACGTAACTTGTTTGATTCGGACCTATCCATCACTATGCCAGACGGACAGCAATTCTCAGTGAATGACCTAAGGGACTTCGATATGTTCCGCATCATGCCAGCGTATGACCGCCGTGTCAATGGTGACATCGCCATCAGGGGGTCTACTGGTAAAACCACTAAGGAACTTAAGGATGAGATTTTGGCTCGCAAAGCGAAAGATGAGGGAGACGGTAAGAAGACTGGCGAGGTACATGCTTTAATGGATACCGGTAAGATTCTTa                                                                                                                                                                   >  1:373968/1‑301 (MQ=255)`  
                                                                                                                                                `gtagGTATCGAGAATAACTCATTCCTTGAGGCACGTAACTTGTTTGATTCGGACCTATCCATCACTATGCCAGACAGACAGCAATTCTCAGTGAATGACCTAAGGGACTTCGATATGTTCCGCATCATGCCAGCGTATGACCGCCGTGTCAATGGTTACATCGCCATCATGGGGTCTACTGGTAAAACCACTAAGGAACTTAAGGATGAGATTTTGGCTCTCAAAGCGAAAGCTGAGGGAGACGGTAAGAAGACTGGCGAGGTACATGCTTTAATGGATACCGTTATGATTCTTACTGGtt                                                                                                                                                             >  1:138102/1‑300 (MQ=255)`  
                                                                                                                                                 `tagGTATCGAGAATAACTCATTCCTTGAGGCACGTAACTTGTTTGATTCGGACCTATCCATCACTATGCCAGACGGACAGCAATTCTCAGTGAATGACCTAAGGGACTTCGATATGTTCCGCATCATGCCAGCGTATGACCGCCGTGTCAATGGTGACATCGCCATCATGGGGTCTACTGGTAAAACCACTAAGGAACTTAATGATGAGATTTTGGCTCTCAAAGCGAAAGCTGAGGGAGACGGTAAGAAGACTGGCGAGGTACATGCTTTAATGGATACCGTTAAGATTCTTACTGGTCg                                                                                                                                                            <  1:405748/301‑1 (MQ=255)`  
                                                                                                                                                      `aTCGAGAATAACTCATTACTTGAGGCACGTAACTTGTTTGATTCGGACCTATCCATCACTATGCCAGACGGACAGCAATTCTCAGTGAATGACCTAAGGGACTTCGATATGTTCCGCATCATGCCAGCGTATGACCGCCGTGTCAATGGTGACATCGCCATCATGGGGTCTACTGGTAAAACCACTAAGGAACTTAATGATGAGATTTTGGCTCTCAAAGCGAAAGCTGAGGGAGACGGTAAGAAGACTGGCGAGGTACATGCTTTAATGGATACCGTTAAGATTCTTACTGGTCGTGCTa                                                                                                                                                       >  1:271063/1‑301 (MQ=255)`  
                                                                                                                                                           `gaATAACTCATTCCTTGAGGCACGTAACTTGTTTGATTCGGACCTATCCATCACTATGCCAGACGGACAGCAATTCTCAGTGAATGACCTAAGGGACTTCGATATGTTCCGCATCATGCCAGCGTATGACCGCCGTGTCAATGGTTACATCGCCATCATGGGGTCTACTGGTAAAACCACTAAGGAACTTAAGGATGAGATTTTGGCTCTCAAAGCGAAAGCTGATGGAGACGGTAAGAAGACTGGCGAGGTACATGCTTTAATGGATACCGTTAAGATTCTTACTGGTctgtctcttata                                                                                                                                                  >  1:104455/1‑290 (MQ=255)`  
                                                                                                                                                                   `cATTCCTTGAGGCACGTAACTTGTTTGATTCGGACCTATCCATCACTATGCCAGACGGACAGCAATTCTCAGTGAATGACCTAAGGGACTTCGATATGTTCCGCATCATGCCAGCGTATGACCGCCGTGTCAATGGTGACATCGCCATCATGGGGTCTACTGGTAAAACCACTAAGGAACTTAAGGATGAGATTTTGGCTCTCAAAGCGAAAGCTGAGGGAGACGGTAAGAAGACTGGCGAGGTACATGCTTTAATGGATACCGTTAAGATTCTTACTGGTCGTGCTactgtctcttatac                                                                                                                                          >  1:278222/1‑288 (MQ=255)`  
                                                                                                                                                                      `gccTTGAGGCACGTAACGTGTTTGATTCGGACCTATCCATCACGATGCCAGACGGACAGCAATTCTCAGTGAATGACCTAAGGGACTTCGATATGTTCCGCATCATGCCAGCGTATGACCGCCGTGTCAATGGTTACATCGCCATCATGGGGTCTACTGGTAAAACCACTAAGGAACTTAAGGATGAGATTTTGGCTCTCAAAGCGAAAGCTGAGGGAGACGGTAAGAAGACTGGCGAGGTACATGCTTTAATGGATACCGTTAAGATTCTTACTGGTCGTGCTAGACGCAATCAGGacac                                                                                                                                       <  1:319778/300‑1 (MQ=255)`  
                                                                                                                                                                            `aGGCACGTAACTTGTTTGATTCGGACCTATCCATCACTATGCCAGACGGACAGCAATTCTCAGTGAATGACCTAAGGGACTTCGATATGTTCCGCATCATGCCAGCGTATGACCGCCGTGTCAATGGTGACATCGCCATCATGGGGTCTACTGGTAAAACCACCAAGGAACTTAAGGATGAGATTTTGGCTCTCAAAGCGAAAGCTGAGGGAGACGGTAAGAAGACTGGCGAGGTACATGCTTTAATGGATACCGTTAAGATTCTTACTGGTCGTGCTAGACGCAATCAGGACACtgtgtg                                                                                                                                 <  1:235636/301‑1 (MQ=255)`  
                                                                                                                                                                                `aCGTAACTTGTTTGATTCGGACCTATCCATCACTATGCCAGACGGACAGCAATTCTCAGTGAATGACCTAAGGGACTTCGATATGTTCCGCATCATGCCAGCGTATGACCGCCGTGTCAATGGTTACATCGCCATCATGGGGTCTACTGGTAAAACCACTAAGGAACTTAAGGATGAGATTTTGGCTCTCAAAGCGAAAGCTGAGGGAGACGGTAAGAAGACTGTCGAGGTACATGCTTTAATGGATACCGTTAAGATTCTTACTGGTCGTGCTAGACGCAATCAGGACACTGTGTGGGaa                                                                                                                             <  1:213769/301‑1 (MQ=255)`  
                                                                                                                                                                                     `aCTTGTTTGATTCGGACCTATCCATCACTATGCCAGACGGACAGCAATTCTCAGTGAATGACCTAAGGGACTTCGATATGTTCCGCATCATGCCAGCGTATGACCGCCGTGTCAATGGTTACATCGCCATCATGGGGTCTACTGGTAAAACCACTAAGGAACTTAAGGATGAGATTTTGGCTCTCAAAGCGAAAGCTGAGGGAGACGGTAAGAAGACTGGCGAGGTACATGCTTTAATGGATACCGTTAAGATTCTTACTGGTCGTGCTAGACGCAATCAGGACACTGTGTGGGAAACCTc                                                                                                                        <  1:274895/301‑1 (MQ=255)`  
                                                                                                                                                                                             `gATTTGGACCTATCCATCACTATGCCAGACGGACAGCAATTCTCAGTGAATGACCTAAGGGACTTCGATATGTTCCGCATCATGCCAGCGTATGACCGCCGTGTCAATGATTACATCGCCATCATGGGGTCTACTGGTAAAACCACTAAGGAACTTAAGGATGAGATTTTGGCTCTCAAAGCGAAAGCTGAGGGAGACGGTAAGAAGACTGGCGAGGTACATGCTTTAATGGATACCGTTAAGATTCTTACTGGTCGTGCTAGACGCACTCAGGACACTGTGTGGGAAAACTCACTGAGTg                                                                                                                >  1:124063/1‑301 (MQ=255)`  
                                                                                                                                                                                             `gATTCGGACCTATCCATCACTATGCCAGACGGACAGCAATTCTCAGTGAATGACCTAAGGGACTTCGATATGTTCCGCATCATGCCAGCGTATGACCGCCGTGTCAATGGTGACATCGCCATCATGGGGTCTACTGGTAAAACCACTAAGGAACTTAAGGATGAGATTTTGGCTCTCAAAGCGAAAGCTGAGGGAGACGGTAAGAAGACTGGCGAGGTACATGCTTTAATGGATACCGTTAAGATTCTTACTGGTCGTGCTAGACGCAATCAGGACACTGTGTGGGAAACCTCACTGCgct                                                                                                                >  1:9473/1‑299 (MQ=255)`  
                                                                                                                                                                                                   `gACCTATCCATCACTATGCCAGACGGACAGCAATTCTCAGTGAATGACCTAAGGGACTTCGATATGTTCCGCATCATGCCAGCGTATGACCGCCGTGTCAATGGTTACATCGCCATCATGGGGTCTACTGGTAAAACAACTAAGGAACTTAAGGATGAGATTTTGGCTCTCAAAGCGAAAGCTGAGGAAGACGGTAAGAAGACTGGCGAGGTACATGCTTTAATGGATACCGGTAAGATTCTTCCTGGTCGTGCTACACGAAATCAGTCCACTGTGTGGGAAACCTCACTGCGTGCCATCa                                                                                                          >  1:17356/1‑301 (MQ=255)`  
                                                                                                                                                                                                     `ccTATCCATCACTATGCCAGACGGACAGCAATTCTCAGTGAATGACCTAAGGGACTTCGATATGTTCAGCATCATGCCAGCGTATGACCGCCGTGTCAATGGTGACATCGCCATCATGGGGTCTACTGGTAAAACCACTAAGGAACTTAAGGATGAGATTTTGGCTCTCAAAGCGAAAGCTGAGGGAGACGGTAAGAAGACTGGCGAGGTACATGCTTTAATGGATACCGTTAAGATTCTTACTGGTCGTGCTAGACGCAATCAGGACACTGTGTGGGAAACCTCACTGCGTGCCATCAAt                                                                                                        >  1:119313/1‑301 (MQ=255)`  
                                                                                                                                                                                                        `aTCCATCACTATGCCAGACGGACAGCAATTCTCAGTGAATGACCTAAGGGACTTCGATATGTTCCTCATCATGCCAGCGTATGACCGCCGTGTCAATGGTGACATCGCCATCATGGGGTCTACTGGTAAAACCACTAAGGAACTTATTGATGAGATTTTGGCTCTCAAAGCGAAAGCTGAGGGAGACGGTAAGAAGACTGGCGAGGTACATGCTTTAATGGATACCGTTAAGATTCTTACTGGTCGTGCTAGACGCAATCAGGACACTGTGTGGGAAACCTCACTGCGTGCCATCAATGAc                                                                                                     >  1:505480/1‑301 (MQ=255)`  
                                                                                                                                                                                                        `aTCCATCACTATGCCAGACGGACAGCAATTCTCAGTGAATGACCTAAGGGACTTCGATATGTTCCGCATCATGCCAGCGTATGACCGCCGTGTCAATGGTTACATCGCCATCATGGGGTCTACTGGTAAAACCACTAAGGAACTTAAGGATGAGATTTTGGCTCTCAAAGCGAAAGCTGAGGGAGACGGTAAGAAGACTGGCGAGGTACATGCTTTAATGGATACCGTTAAGATTCTTACTGGTCGTGCTAGACGCACTCAGGACACTGTGTGGGCAAccctgtttcttatcaccattccc                                                                                                     >  1:287257/1‑280 (MQ=255)`  
                                                                                                                                                                                                        `aTCCATCACTATGCCAGACGGACAGCAATTCTCAGTGAATGACCTAAGGGACTTCGATATGTTCCGCATCATGCCAGCGTATGACCGCCGTGTCAATGGTGACATCGCCATCATGGGGTCTACTGGTAAAACCACTAAGGAACTTAAGGATGAGATTTTGGCTCTCAAAGCGAAAGCTGAGGGAGACGGTAAGAAGACTGGCGAGGTACATGCTTTAATGGATACCGTTAAGATTCTTACTGGTCGTGCTAGACGCAATCAGGACACTGTGTGGGCAACCTCAATGctgtctcttatacac                                                                                                     >  1:314504/1‑287 (MQ=255)`  
                                                                                                                                                                                                        `aTCCATCACTATGCCAGACGGACAGCAATTCTCAGTGAATGACCTAAGGGACTTCGATATGTTCCGCATCATGCCAGCGTATGACCGCCGTATCAATGGTGACATCGCCATCATGGGGTCTACTGGTAAAACCACTAAGGAACTTAATGATGAGATTTTGGCTCTCAAAGCGAAAGCTGAGGGAGACGGTAAGAAGACTGGCGAGGTACATGCTTTAATGGATACCGTTAAGCTTCTTACTGGTCGTGCTAGACGCAATCAGGACACTGTGTGGGAAACCTCACTGCGTGccctgtctctt                                                                                                     >  1:42185/1‑292 (MQ=255)`  
                                                                                                                                                                                                         `tCCATCACTATGCCAGACGGACAGCAATTCTCAGTGAATGACCTAAGGGACTTCGATATGTTCCGCATCATGCCAGCGTATGACCGCCGTGTCAATGGTTACATCGCCATCATGGGGTCTACTGGTAAAACCACTAAGGAACTTAAGGATGAGATTTTGGCTCTCAAAGCGAAAGCTGAGGGAGACGGTAAGAAGACTGGCGAGGTACATGCTTTAATGGATACCGTTAAGATTCTTACTGGTCGTGCTAGACGCAATCAGGACACTGTGTGGGAAACCTCACTGCGTGCCATCAATGAcc                                                                                                    >  1:337203/1‑301 (MQ=255)`  
                                                                                                                                                                                                          `ccATCACTATGCAAGACGGACAGCAATTCTCAGTGAATGACCTAAGGGACTTCGATATGTTCCGCATCTTGCCAGCGTATGACCGCCGTGTCAATGGTGACATCGCCATCATGGGGTCTACTGGTAAAACCACTAAGGAACTTAATGATGAGATTTTGGCTCTCAAAGCGAAAGCTGATGGAGACGGTAAGAAGACTGGCGAGGTACATGCTTTAATGGATACCGTTAAGATTCTTACTGGTCGTGCTAGACGCAATCAGGACACTGTGTGGGAAACCTCACTGCGTGCCATCAATGACCt                                                                                                   <  1:409709/301‑1 (MQ=255)`  
                                                                                                                                                                                                              `cACTATGCCAGACTGACAGCAATTCTCAGTGAATGACCTAAGGGACTTCGATATGTTCCGCATCATGCCAGCGTATGACCGCCGTGTCAATGGTTACATCGCCATCATGGGGTCTACTGGTAAAACCACTAAGGAACTTAAGGATGAGATTTTGGCTCTCAAAGCGAAAGCTGAGGGAGACGGTAAGAAGACTGGCGAGGTACATGCTTTAATGGATACCGTTAAGATTCTTACTGGTCGTGCTAGACGCAATCAGGACACTGTGTGGGAAACCTCACTGCGTGCCATCAATGACCTAggg                                                                                               <  1:378489/301‑1 (MQ=255)`  
                                                                                                                                                                                                              `cACTATGCCAGACGTACAGCAATTCTCAGTGAATGACCTAAGGGACTTCGATATATTCCGCATCATGCCAGCGTATGACCGCCGTGTCAATGGTGACATCGCCATCATGGGGTCTACTGGTAAAACCACTAAGGAACTTAATGATGAGATTTTGGCTCTCAAAGCGAAAGCTGAGGGAGACGGTAAGAAGACTGGCGAGGTACATGCTTTAATGGATACCGTTAAGATTCTTACTGGTCGTGCTAGACGCAATCAGGACACTGTGTGGGAAACCTCACTGCGTGCCATCAATGACCTAggg                                                                                               <  1:19202/301‑1 (MQ=255)`  
                                                                                                                                                                                                              `cACTATGCCAGACGGACAGCAATTCTCAGTGAATGACCTAAGGGACTTCGATATGTTCCGCATCATGCCAGCGTATGAACGCCGTGTCAATGGTTACATCGCCATAATGGGGTCTAATGGTAAAACAACTAAGGAACTTAAGGATGAGATTTAGGCTCTCAAAGCGAAAGCTGAGGGAGACGGTAAGAAGACTGGAGAGGTACATGCTTTAATGGATACCGTTAAGATTCTTACTGGTCGTGCTAGACGCCATAAGGACACTGTGTGGGAAAccctgtatcttttacacatctccgagccc                                                                                               >  1:510349/1‑274 (MQ=255)`  
                                                                                                                                                                                                              `cACTATGCCAGACGGACAGCAATTCTCAGTGAAAGACCTAAGGGACTTCGATATGTACTGCATCATGCCAGCGTATGACCGCCGTGTCAATGGTTACATCGCCATCATGGGGTCTACTGGTAAAACCACTAAGGAACTTAAGGATGAGATTTTGGCTCTCAAAGCGAAAGCTGAGGGAGACGGTAAGAAGACTGGCGAGGTACATGCTTTAATGGATACCGTTAAGATTCTTACTGGTCGTGCTAGACGCAATCAGGACACTGTGTGGGAAACCTCACTGCGTGCCATCAATGACCTAggg                                                                                               <  1:383100/301‑1 (MQ=255)`  
                                                                                                                                                                                                                `cTATGCCAGACGGACAGCAATTCTCAGTGAATGACCTAAGGGACTTCGATATGTTCCGCATCATGCCAGCGTAGGACCGCCGTGTAAATGGTGACATCGCCATCATGGGGTCTACTGGTAAAACCACTAAGGAAGTTAATGATGAGATTTTGGCTCTCAAAGCGAAAGCTGAGGGAGACGGTAAGAAGTCTGGCGAGGTACATGCTTTAATGGATACCGTTAAGATTCTTACTGGTCGTGCTAGACGCAATCAGGACACTGTGTGGGAAACCTCAcctgtctatgatacacatctccgagc                                                                                             >  1:448343/1‑276 (MQ=255)`  
                                                                                                                                                                                                                  `aTGCCAGACGGACAGCAATTCTCAGTGAATGACCTAAGGGACTTCGATATGTTCCGCATCATGCCAGCGTATGACCGCCGTGTCAATGGTGACATCGCCATCATGGGGTCTACTGGTAAAACCACTAAGGAACTTAATGATGAGATTTTGGCTCTCAAAGCGAAAGCTGAGGGAGACGGTAAGAAGACTGGCGAGGTACATGCTTTAATGGATACCGTTAAGATTCTTACTGGTCGTGCTAGACGCAATCAGGACACTGTGTGGGAAACCTCACTGCGTGCCATCAATGACCTAGGGttct                                                                                           >  1:698/1‑301 (MQ=255)`  
                                                                                                                                                                                                                      `cAGACGGACAGCAATTCTCAGTGAATGACCTAAGGGACTTCGATATGTTCCGCATCATGCCAGCGTATGACCGCCGTGTCAATGGTTACATCGCCATCATGGGGTCTACTGGTAAAACCACTAAGGAACTTAAGGATGAGATTTTGGCTCTCAAAGCTAAAGCTGAGGGAGACGGTAAGAAGACTGGCGAGGTACATGCTTTAATGGATACCGTTAAGATTCTTACTGGTCGTGCTAGACGCAATCAGGACACTGTGTGGGAAACCTCACTGCGTGCCATCAATGACCTAGGGTTCTTCGc                                                                                       <  1:413796/301‑1 (MQ=255)`  
                                                                                                                                                                                                                           `ggACAGCAATTCTCAGTGAATGACCTAAGGGACGTCGATATGTTCCGCATCATGCCAGCGTATGAACGACGTGTCAATGGTGACATCGCCATAATGGGGTCTACTGGTAAAACCACTAAGGAACTTAAGGATGAGATTTTGGCTCTAAAAGCGAAAGCTGAGGGAGACGGTAAGAAGACTGGCGAGGTACATGCTTTAATGGATACCGTTAAGATTCTTACTGGTCGTGCTAGACGCAATCAGGACACTGTGTGGGAAACCTCACTGCGTGCCATCAATGACCTAGGGTTCTTCGCTAAGa                                                                                  <  1:462219/301‑1 (MQ=255)`  
                                                                                                                                                                                                                                     `tctcAGTGAATGACCTAAGGGACTTCGATATGTTCCGCATCATGCCAGCGTATGACCGCCGTGTCAATGGTGACATCGCCATCATGGGGTCTACTGGTAAAACCACTAAGGAACTTAATGATGAGATTTTGGCTCTCAAAGCGAAAGCTGAGGGAGACGGTAAGAAGACTGGCGAGGTACATGCTTTAATGGATACCGTTAAGATTCTTACTGGTCGTGCTAGACGCAATCAGGACACTGTGTGGTAAACCTCACTGCGTGCCATCAATGACCTAGGGttcctttcccttatacacatctc                                                                        >  1:196247/1‑281 (MQ=255)`  
                                                                                                                                                                                                                                     `aataaggaacagACCAAAGGGACTTAGATATGTTCAGCATCATGCCAGCGGATGACTGCCGTGTCAATGGTTACATCGCCATCATGGGGTCTACTGGTAAAACCACTAAGGAACGTAAGGATGAGATATTGGCTATCAGAGCGAAAGCTGAGGGAGACGGTAAGAAGACTGGCGAGGTACATGCTTTAATGGATACCGTTAAGATTCTTACTGGTCGTGCTAGACGCAATCAGGACACTGTGTGGGAAACCTCACTGCGTGCCATCAATGACCTAGGGTTCTTCGCTAAGAACGCCTACAt                                                                        <  1:396679/290‑1 (MQ=255)`  
                                                                                                                                                                                                                                      `ctcAGTGAATGACCTAAGGGACTTCGATATGTTCCGCATCATGCCAGCGTATGACCGCCGTGTCAATGGTTACATCGCCATCATGGGGTCTACTGGTAAAACCACTAAGGAACTTAAGGATGAGATTTTGGCTCTCAAAGCGAAAGCTGAGGGAGACGGTAAGAAGACTGGCGAGGTACATGCTTTAATGGATACCGTTAATATTCTTACTGGTCGTGCTAGACTCAATCAGGACACTGTGTGGGAAACCTCACTGCGTGCCATCAATGACCTAGGGTTCTTCGCTAAGAACGCCTACATg                                                                       <  1:18177/301‑1 (MQ=255)`  
                                                                                                                                                                                                                                          `gagAATGACCCAAGGGACTTCGATATGTTCCGCATCATGCCAGAGTAGGACCGCCGTGTCAATGGTTACATCGCCATCATGGGGTCTACTGGTAAAACCACTAAGGAACTTAAGGATGAGATTTTGGCTCTAAAAGCGAAAGCTGAGGGAGACGGTAAGAAGACTGGCGAGGTACATGCTTTAATGGATACCGTTAAGATTCTTACTGGTCGTGCTAGACGCAATCAGGACACTGTGTGGGAAACCTCACTGCGTGCCATCAATGACCTAGGGTTCTTCGCTAAGAACGCCTACATGGGTg                                                                   <  1:33351/299‑1 (MQ=255)`  
                                                                                                                                                                                                                                          `gTGAATGACCTAAGGGACTTCGATATGTTCCGCATCATGCCAGCGTATGACCGCCGTGTCAATGGTGACATCGCCATCATGGGGTCTACTGGTAAAACCACTAAGGAACTTAAGGATGAGATTTTGGCTCTCAAAGCGAAAGCTGAGGGAGACGGTAAGAAGACTGGCGAGGTACATGCTTTAATGGATACCATTAAGATTCTTACTGGTCGTGCTAGACGCAATCAGGACACTGTGTGGGAAACCTCACTGCGTGCCATCAATGACCTAGGGTTCTTCGCTAAGAACGCCTACATGGGTg                                                                   >  1:262871/1‑301 (MQ=255)`  
                                                                                                                                                                                                                                          `gTGAATGACCTAAGGGACTTCGATATGTTCCGCATCATGCCAGCGTATGACCGCCGTGTCAATGGTGACATCGCCATCATGGGGTCTACTGGTAAAACCACTAA‑GTACTTAATGATGAGATTTTGGCTCTCAAAGCGCAAGCTGAGGTAGACGGTAAGAAGACTGGCGAGGTACATGCTTTAATGGATACCGTTAAGATTCTTACTGGTCGTGCTAGACGCAATCAGGACACTGTGTGGGAAACCTCACTGCGTGCCATCAATGACCTAGGGTTCTTCGCTAAGAACGCCTACATGGGTGc                                                                  >  1:231028/1‑301 (MQ=255)`  
                                                                                                                                                                                                                                             `aaTGACCTAAGGGACTTCGATATGTTCCGCATCATGCCAGCGTATGACCGCCGTGTCAATGGTTACATCGCCATCATGGGGTCTACTGGTAAAACCACTAAGGAACTTAAGGATGAGATTTTGGCTCTCAAAGCGAAAGCTGAGGGAGACGGTAAGAAGACTGGCGAGGTACATGCTTTAATGGATACCGTTAAGATTCTTACTGGTCGTGCTAGACGCAATCAGGACACTGTGTGGGAAACCTCACTGCGTGCCATCAATGACCTAGGGTTCTTCGCTAAGAACGCCTACATGGGTGCTc                                                                <  1:342652/301‑1 (MQ=255)`  
                                                                                                                                                                                                                                               `tGACCTAAGGGACTTCGATATGTTCCGCATCATGCCAGCGTATGACCGCCGTGTCAATGGTTACATCGCCATCATGGTGTCTACTGGTAAAACCACTAAGGAACTTAAGGATGAGATTTTGGCTCTCAAAGCGAAAGCTGAGTGAGACGGTAAGAAGACTGGCGAGGTACATGCTTTAATGGATACCGTTAAGATTCTTACTGGTCGTGCTAGACGCAATCAGGACACTGTGTGGGAAACCTCACTGCGTGCCATCAATGACCTAGGGTTCTTCGCTAAGAACGCCTACATGGGTGCTCAg                                                              <  1:50045/301‑1 (MQ=255)`  
                                                                                                                                                                                                                                                 `aCCTAAGGGACTTCGATATGTTCCGCATCATGCCAGCGTATGACCGCCGTGTCAATGGTTACATCGCCATCATGGGGTCTACTGGTAAAACCACTAAGGAACTTAAGGATGAGATTTTGGCTCTCAAAGCGAAAGCTGAGGGAGACGGTAAGAAGACTGGCGAGGTACATGCTTTAATGGATACCGTTAAGATTCTTACTGGTCGTGCTAGACGCAATCAGGACACTGTGTGGGAAACCTCACTGCGAGCCATCAATGACCTAGGGTTCTTCGATAAGAGCGCCTACATGGGTACTCAGaa                                                            >  1:209025/1‑301 (MQ=255)`  
                                                                                                                                                                                                                                                 `aCCTAAGGGACTTCGATATGTTCCGCATCATGCCAGCGTATGACCGCCGTGTCAATGGTGACATCGCCATCATGGGGTCTACTGGTAAAACCACTAAGGAACTTAATGATGAGATTTTGGCTCTCAAAGCGAAAGCTGAGGGAGACGGTAAGAAGACTGGCGAGGTACATGCTTTAATGGATACCGTTAAGATTCTTACTGGTCGTGCTAGACGTAATCAGGACACTGTGTGGGAAACCTCACTGCGTGCCATCAATGACCTAGGGTTCTTCGCTAAGAACGCCTACAtctgtctcttata                                                            >  1:132734/1‑289 (MQ=255)`  
                                                                                                                                                                                                                                                 `aCCTAAGGGACTTCGATATGTTCCGCATCATGCCAGCGTATGACCGCCGTGTCAATGGTGACATCGCCATCATGGGGTCTACTGGTAAAACCACTAAGGAACTTAATGATGAGATTTTGGCTCTCAAAGCGAAAGCTGAGGGAGACGGTAAGAAGACTGGCGAGGTACATGCTTTAATGGATACCGTTAAGATTCTTACTGGTCGTGCTAGACGCAATCAGGACACTGTGTGGGAAACCTCACTGCGTGCCATCAATGACCTAGGGTTCTTCGCTAAGAACGCCTACATGGGTGCTCAGaa                                                            >  1:156300/1‑301 (MQ=255)`  
                                                                                                                                                                                                                                                 `aCCTAAGGGACTTCGATATGTTCCGCATCATGCCAGCGTATGACCGCCGTGTCAATGGTGACATCGCCATCATGGGGTCTACTGGTAAAACCACTAAGGAACTTAAGGATGAGATTTTGGCTCTCAAAGCGAAAGCTGAGGGAGACGGTAAGAAGACTGGCGAGGTACATGCTTTAATGGATACCGTTAAGATTCTTACTGGTCGTGCTAGACGCAATCAGGACACTGTGTGGGAAACCTCACTGCGTGCCATCAATGACCTAGGGTTCTTCGCTAAGAACGCCTACATGGGTGCTCAgta                                                            >  1:439534/1‑299 (MQ=255)`  
                                                                                                                                                                                                                                                  `aagagacagcTTCGATATGTTCCGCATCATGCCAGCGTATGACCGCCGTGTCAATGGTTACATCGCCATCATGGGGTCTACTGGTAAAACCACTAAGGAACTTAAGGATGAGATTTTGGCTCTCAAAGCGAAAGCTGAGGGAGACGGTAAGAAGACTGGCGAGGTACATGCTTTAATGGATACCGTTAAGATTCTTACTGGTCGTGCTAGACGCAATCAGGACACTGTGTGGGAAACCTCACTGCGTGCCATCAATGACCTAGGGTTCTTCGCTAAGAACGCCTACATGGGTGCTCAGAAc                                                           <  1:444038/292‑1 (MQ=255)`  
                                                                                                                                                                                                                                                     `aaGGGACTTCGATATGTTCCGCATCATGCCAGCGTATGACCGCCGTGTCAATGGTTACATCGCCATCATGGGGTCTACTGGTAAAACCACTAAGGAACTTAAGGATGAGATTTTGGCTCTCAAAGCGAAAGCTGAGGGAGACTGTAAGAAGACTGGCGAGGTACATGCTTTAATGGATACCGTTAAGATTCTTACTGGTCGTGCTAGACGCAATCAGGACACTGTGTGGGAAACCTCACTGCGTGCCATCAATGACCTAGGGTTCTTCGCTAAGAACGCCTACATGGGTGCTCAGAACAtt                                                        <  1:85639/301‑1 (MQ=255)`  
                                                                                                                                                                                                                                                       `gggACTTCGATATGTTCCGCATCATGCCAGCGTATGACCGCCGTGTCAATGGTGACATCGCCATCATGGGGTCTACTGGTAAAACCACTAAGGAACTTAAGGATGAGATTTTGGCTCTCAAAGCGAAAGCTGAGGGAGACGGTAAGAAGACTGGCGAGGTACATGCTTTAATGGATACCGTTAAGATTCTTACTGGTCGTGCTAGACGCAATCAGGACACTGTGTGGGAAACCTCACTGCGTGCCATCAATGACCTAGGGTTCTTCGCTAAGAACGCCTACATGGGTGCTCAGAACATTAc                                                      <  1:186658/301‑1 (MQ=255)`  
                                                                                                                                                                                                                                                       `gcggagatgtgtatgagaaacagatGCCAGCGTATGACCTCCGTGTCAATGGTGACATCGCCATCATGGGGTCTACTGGGAAAACCACTAAGGAACTTAAGGATGAGATTTTGGCTCTCAAAGCGAAAGCTGAGGGAGACGGTAAGAAGACTGGCGAGGTACATGCTTTAATGGATACCGTTAAGATTCTTACTGTTCGTGCTAGACTCAATCAGGACACTGTGTGGGAAACCTCGCTGCGTGCCATCAATGACCTATGGTTCTTCGCTAAGAACGCCTACATGGGTGCTCAGAACATTAc                                                      <  1:159752/278‑1 (MQ=255)`  
                                                                                                                                                                                                                                                         `ataagagccaggTTCCGCCTCATGCCAGCGTATGACCGCCGGTTCAATGGGTACATCGCCATCATGGGGGTTAATGGTAAAACCCCTAAGGAACTTAAGGATGAGATTTTGGCTCTCAAAGCGAAAGCTGAGGGAGACGGTAAGAAGACTGGCGAGGTACATGCTTTAATGGATACCGTTAAGATTCTTACTGGTCGTGCTAGACGCAATCAGGACACTGTGTGGGAAACCTCACTGCGTGCCATCAATGACCTAGGGTTCTTCGCTAAGAACGCCTACATGGGTGCTCAGAACATTACgg                                                    <  1:258244/290‑1 (MQ=255)`  
                                                                                                                                                                                                                                                           `tttCCATGTGTTCCGCAACATGCCAGCGTAAGACCGCCGTGTCAATGGTTACATCGCCATCATGGGGTCTACTGGTAAAACCAATAAGGAACTTAAGGATGAGATTTTTGCTCTCAAAGCGGAAGCTGAGGGAGACGGTAAGAAGACTGGCGAGGTACATGCTTTAATGGATACCGTTAAGATTCTTACTGGTCGTGCTAGACGCAATCAGAACACTGTGTGGGAAACCTCACTGCGTGCCATCAATGACCTAGGGTTCTTCGCTAAGAACGCCTACATTGGTGCTCAGAACATTACGgag                                                  <  1:391892/300‑1 (MQ=255)`  
                                                                                                                                                                                                                                                           `cTTCGATATGTTCCGCATCATGCCAGCGTATGACCGCCGTGTCAATGGTTACATCGCCATCATGGGGTCTACTGGTAAAACCACTAAGGAACTTAAGGATGAGATTTTGGCTCTCAAAGCGAAAGCTGAGGGAGACGGTAAGAAGACTGGCGAGGTACATGCTTTAATGGATACCGTTAAGATTCTTACTGGTCGTGCTAGACGCAATCAGGACACTGTGTGGGAAACCTCACTGCGTGCCATCAATGACCTAGGGTTCTTCGCTAAGAACGCCTACATGGGTGCTCAGAAAATTAcctgt                                                  >  1:221857/1‑297 (MQ=255)`  
                                                                                                                                                                                                                                                             `tCGATATGTTCCGCATCATGCCAGCGTATGACCGCCGTGTCAATGGTGACATCGACATCATGGGGTCTACTGGTAAAACCACTAAGGAACTTAATGATGAGATTTTGGCTCTCAAAGCGAAAGCTGAGGGAGACGGTAAGAAGACTGGCGAGGTACATGCTTTAATGGATACCGTTAAGATTCTTACTGGTCGTGCTAGACGCAATCAGGACACTGTGTGGGAAACCTCACTGCGTGCCATCAATGACCTAGGGTTCTTCGCTAAGAACGCCTACATTGGTGCTCAGAACTTTACGGAGAt                                                >  1:488586/1‑301 (MQ=255)`  
                                                                                                                                                                                                                                                              `cGATATGTTCCGCATCATGCCAGCGTATGACCGCCGTGTCAATGGTTACATCGCCATCATGGGGTCTACTGGTAAAACCACTAAGGAACTTAAGGATGAGATTTTGGCTCTCAAAGCGAAAGCTGAGGGAGACGGTAAGAAGACTGGCGAGGTACATGCTTTAATGGATACCGTTAAGATTCTTACTGGTCGTGATTGACGCAATCAGGACACTGTGTGGGAAACCTCACTGCGTGCCATCAATGACCTAGGGTTCTTCGCTAAGAACGCCTACAAGGGTGCCCAGAAAATAAAGGAGAtt                                               >  1:110957/1‑301 (MQ=255)`  
                                                                                                                                                                                                                                                                   `tGTTCCGCATCATGCCAGCGTATTACCGCCGTGTCAATGTTGACATCGCCATCATGGGGTCTACTGGTAAAACCACTAAGGAACTTAATGATGAGATTTTGGCTCTCAAAGCGAAAGCTGAGGGAGACGGTAAGAAGACTGGCGAGGTACATGCTTTAATGGATACCGTTAAGATTCTTACTGGTCGTGCTAGACGCAATCAGGACACTGTGTGGGAAACCTCACTGCGTGCCATCAATGACCTAGGGTTCTTCGCTAAGAACGCCTACATGGGTGCTCAGAACATTACGGAGATTGCTgg                                          <  1:483498/301‑1 (MQ=255)`  
                                                                                                                                                                                                                                                                     `ttCCGCATCATGCCAGCGTATGACCGCCGTGTCAATGGTGACATCGCCATCATGGGGTCTACTGGTAAAACCACTAAGGAACTTAAGGATGAGATTTTGGCTCTCAAAGCGAAAGCTGAGGGAGACGGTAAGAAGACTGGCGAGGTACATGCTTTAATGGATACCGTTAAGATTCTTACTGGTCGTGCTAGACGCAATCAGGACACTGTGTGGGAAACCTCACTGCGTGCCATCAATGACCTAGGGTTCTTCGCTAAGAACGCCTACATgggctgtctcttataaacatctccgagcccac                                        >  1:227616/1‑272 (MQ=255)`  
                                                                                                                                                                                                                                                                               `tGCCAGCGTATGACCGCCGTGTCAATGGTGACATCGCCATCATGTGGTCTACTGGTAAAACCACTAAGGAACTTAATGATGAGATTTTGGCTCTAAAAGCGAAAGCTGAGGGAGACGGTAAGAAGACTGGCGAGGTACATGCTTTAATGGATACCGTTAAGATTCTTACTGGTCGTGCTAGACGCAATCAGGACACTGTGTGGGAAACCTCACTGCGTGCCATCAATTACCTAGGGTTCTTCGCTAAGAACGCCTACATGGGTGCTCAGAAGATTACGGAGATTGCTGGGATGATTGTCAc                              <  1:64795/301‑1 (MQ=255)`  
                                                                                                                                                                                                                                                                                    `gCGTATGACCGCCGTGTCAATGGTGACATCGCCATCATGGGGTCTACTGGTAAAACCACTAAGGAACTTAAGGATGAGATTTTGGCTCTCAAAGCGAAAGCTGATGGAGACGGTAAGAAGACTGGCGAGGTACATGCTTTAATGGATACCGTTAAGATTCTTACTGGTCGTGCTAGACGCAATCCGGACACTGTGTGGGAAACCTCACTGACTGCCATCAATGACCTAGGGTTCTTCGCTAATAACGCCTACATGGGTTCTCAGAACATTACGGCGCTTGCTGGGACGTTTGttcatggta                         >  1:304789/1‑293 (MQ=255)`  
                                                                                                                                                                                                                                                                                      `gTATGACCGCCGTGTCAATGGTGACATCGCCATCATGGGGTCTACTGGTAAAACCACTAAGGAACTTAAGGATGAGATTTTGGCTCTCAAAGCGAAAGCTGAGGGAGACGGTAAGAAGACTGGCGAGGTACATGCTTTAATCGATACCGTTAAGATTCTTACTGGTCGTGCTAGACGCAATCAGGACACTGTGTGGGAAACCTCACTGCGTGCCATCAATGACCTAGGGTTCTTCGCTAAGAACGCCTACATGGGTGCTCAGAACATTACGGAGATTGCTGGGATGATTGTCACTGGTAAc                       >  1:16412/1‑301 (MQ=255)`  
                                                                                                                                                                                                                                                                                                  `tgtCAATGGTTACATCGCCATAATGGGGTCTACTGGTAAAACCACTAAGGAACTTAAGGATGAGATTTTGGCTCTCAAAGCGAAAGCTGAGGGAGACGGTAAGAAGACTGGCGAGGTACATGCTTTAATGGATACCGTTAAGATTCTTACTGGTCGTGCTAGACGCAATCAGGACACTGTGTGGGAAACCTCACTGCGTGCCATCAATGACATAGGGTTCTTCGCTAAGAACGCCTACATGGGTGCTAAGAACATTACGGAGATTGCTGGGATGATTGTCACTGGTAACGTTCGTGCTCTa           >  1:201535/1‑301 (MQ=255)`  
                                                                                                                                                                                                                                                                                                      `aaTGGTGACATCGCCATCATGGGGTCTACTGGTAAAACCACTAAGGAACTTAATGATGAGATTTTGGCTCTCAAAGCGAAAGCTGAGGGAGACGGTAAGAAGACTGGCGAGGTACATGCTTTAATGGATACCGTTAAGATTCTTACTGGTCGTGCTAGACGCAATCAGGACACTGTGTGGGAAACCTCACTGCGTGCCATCAATGACCTAGGGTTCTTCGCTAAGAACGCCTACATGGTTGCTCAGAACATTACGGAGATTGCTAGTATGATTGTCACTGGTAACGTTCGTGCTCTAGGGc       >  1:292132/1‑301 (MQ=255)`  
                                                                                                                                                                                                                                                                                                         `ggTTACATCGCCATCATGGGGTCTACTGGTAAAACCACTAAGGAACTTAAGGATGAGATTTTGGCTCTCAAAGCGAAAGCTGAGGGAGACGGTAAGAAGACTGGCGAGGTACATGCTTTAATGGTTACCGTTAAGATTCTTACTGGTCGTGCTAGACGCAATCAGGACACTGTGTGGGAAACCTCACTGCGTGCCATCAATGACCTAGGGTTCTTCGCTAAGAACGCCTACATGGGTGCTCAGAACATTACGGAGATTGCTGGGATGATTGTCACTGGTAACGTTCTTGCTCTAgctgtct    >  1:454523/1‑295 (MQ=255)`  
                                                                                                                                                                                                                                                                                                           `tGACATCGCCATCATGGGGTCTACTGGTAAAACCACTAAGGAACTTAAGGATGAGATTTTGGCTCTCAAAGCGAAAGCTGAGGGAGACGGTAAGAAGACTGGCGAGGTACATGCTTTAATGGATACCGTTAAGATTCTTACTGGTCGTGCTAGACGCAATCAGGACACTGTGTGGGAAACCTCACTGCGTGCCATCAATGACCTAGGGTTCTTCGCTAAGAACGCCTACATGGGTGCTCAGAACATTACGGAGATTGCTGGGATGATTGTCACTGGTAACGTTCGTGCTCTAGGGCATGGt  <  1:471014/301‑1 (MQ=255)`  

                                                                                                                                                                                                                                                                                                            `|`                                                                                                                                                                                                                                                                                                             
`GATGAGATGCTTAAGGAATTACACGGGGTGAAGGAAGTAACACCAGAGATGGTAGAGAAGTACGCTATGGATAAGGCTTATGGTATCTCCCACTCAGACCAGTTCACCAACAGTTCCATAATAGAAGAGAACATTGAGGGCTTAGTAGGTATCGAGAATAACTCATTCCTTGAGGCACGTAACTTGTTTGATTCGGACCTATCCATCACTATGCCAGACGGACAGCAATTCTCAGTGAATGACCTAAGGGACTTCGATATGTTCCGCATCATGCCAGCGTATGACCGCCGTGTCAATGGTGACATCGCCATCATGGGGTCTACTGGTAAAACCACTAAGGAACTTAAGGATGAGATTTTGGCTCTCAAAGCGAAAGCTGAGGGAGACGGTAAGAAGACTGGCGAGGTACATGCTTTAATGGATACCGTTAAGATTCTTACTGGTCGTGCTAGACGCAATCAGGACACTGTGTGGGAAACCTCACTGCGTGCCATCAATGACCTAGGGTTCTTCGCTAAGAACGCCTACATGGGTGCTCAGAACATTACGGAGATTGCTGGGATGATTGTCACTGGTAACGTTCGTGCTCTAGGGCATGGT  >  NC_001604.1/32722‑33321`

  

Alignment Legend

Aligned base mismatch/match (shaded by quality score): `ATCG/ATCG` `< 3 ≤` `ATCG/ATCG` `< 10 ≤` `ATCG/ATCG` `< 18 ≤` `ATCG/ATCG` `< 34 ≤` `ATCG/ATCG` `< 38 ≤` `ATCG/ATCG`

Unaligned base: `atcg`    Masked matching base: `atcg`    Alignment gap: `‑`    Deleted base: `‑`
